# Plank Times and Lower Extremity Overuse Injury in Collegiate Track-and-Field and Cross Country Athletes

**DOI:** 10.3390/sports10030045

**Published:** 2022-03-21

**Authors:** Lace E. Luedke, Mitchell J. Rauh

**Affiliations:** 1 Department of Kinesiology, University of Wisconsin Oshkosh, Oshkosh, WI 54901, USA; 2 Doctor of Physical Therapy Program, San Diego State University, San Diego, CA 92182, USA; mrauh@sdsu.edu

**Keywords:** core endurance, lumbopelvic stability, running-related injury

## Abstract

Trunk muscle endurance has been theorized to play a role in running kinematics and lower extremity injury. However, the evidence examining the relationships between static trunk endurance tests, such as plank tests, and lower extremity injury in athletes is conflicting. The purpose of this study was to assess if collegiate cross country and track-and-field athletes with shorter pre-season prone and side plank hold times would have a higher incidence of lower extremity time-loss overuse injury during their competitive sport seasons. During the first week of their competitive season, 75 NCAA Division III uninjured collegiate cross country and track-and-field athletes (52% female; mean age 20.0 ± 1.3 years) performed three trunk endurance plank tests. Hold times for prone plank (PP), right-side plank (RSP) and left-side plank (LSP) were recorded in seconds. Athletes were followed prospectively during the season for lower extremity overuse injury that resulted in limited or missed practices or competitions. Among the athletes, 25 (33.3%) experienced a lower extremity overuse injury. There were no statistically significant mean differences or associations found between PP, RSP or LSP plank test hold times (seconds) and occurrence of lower extremity overuse injury. In isolation, plank hold times appear to have limited utility as a screening test in collegiate track-and-field and cross country athletes.

## 1. Introduction

Across National Collegiate Athletic Association NCAA divisions, collegiate track-and-field and cross country are among the sports with the most participants with over 59,000 and 29,000 student-athletes competing, respectively [1]. However, recent research indicates that NCAA cross country and track-and-field athletes experience a higher rate of overuse injury relative to other sports [2].

Reduced trunk muscle endurance has been associated with lower extremity injuries and lower-back pain in athletes from several sports [3,4,5]. For example, Wilkerson et al. observed that shorter hold times for anterior trunk muscles, using a trunk flexion hold, were associated with increased risk of lower extremity injuries in collegiate football players [3,4]. Shorter hold times for trunk muscle endurance, assessed with side planks, anterior and posterior trunk tests, have been associated with increased incidence of lower-back pain among male collegiate athletes competing in soccer, basketball, handball and volleyball [5].

Clinically, trunk muscle endurance can be measured by using prone and side plank tests. The prone plank is a valid and reliable assessment of anterior abdominal muscular endurance [6]. In active collegiate physical education students, shorter prone plank times were associated with a higher risk of lower extremity injury [7]. De Blaiser et al. found the prone plank test to be predictive of lower extremity overuse injury in active collegiate physical-education students. They observed that every 1 s decrease in the prone bridging (plank) times increased the risk of injury by 1% [7]. They also reported that prone plank and side plank mean hold times were significantly shorter in students who experienced overuse injuries [7].

The side plank test requires high levels of muscular activity of the external obliques, quadratus lumborum and gluteus medius [8,9]. While the side plank test is considered a safe and reliable measure of lateral trunk musculature [9], its use as a predictor of injury in runners is not as clear. In recreational runners, shorter side plank hold times have been significantly correlated with increased peak hip internal rotation during running, with side plank times accounting for 12.7% of the variability in internal rotation [10]. Thus, this suggests that the side plank test may be of value in runners, as increased hip internal rotation during running has been associated with common injuries, such as patellofemoral pain syndrome [11]. Conversely, while Leetun et al. observed that side plank hold times were significantly correlated with hip abduction and external rotation strength measures in collegiate basketball and track athletes, they reported that side plank hold times were not significantly shorter in the athletes who experienced lower extremity injuries during their season compared to those who did not incur a lower extremity injury [12]. Furthermore, a recent systematic review indicated conflicting evidence for using trunk endurance tests for injury screening in healthy athletic populations, as some studies have found relationships between trunk muscle endurance and injury risk while others have not [13].

Trunk muscle endurance exercises, including front and side planks, are often included in rehabilitation programs for common running injuries [14,15,16,17,18], as well as in programs developed to prevent running-related injuries [19,20] or improve performance [21]. However, the current evidence on their validity as a screening tool for injury risk is inconsistent. Thus, the purpose of this prospective study was to determine whether prone and side plank hold times were associated with lower extremity time-loss overuse injury in NCAA track-and-field and cross country athletes. We hypothesized that shorter plank times would be associated with a higher incidence of lower extremity time-loss overuse injury during their intercollegiate seasons.

## 2. Materials and Methods

Participants: Seventy-five (39 females, 36 males) NCAA Division III collegiate cross country and track-and-field athletes participated in the study. Eight athletes were unable to participate due to not being cleared for full participation at the start of the season. The study was approved by the university’s Institutional Review Board, and informed consent was obtained from all athletes prior to study participation.

Questionnaire: During the first week of the competitive season, the participants completed the study questionnaire which inquired about their age, body mass, height and prior injury history.

Trunk Muscle Endurance Assessment: After completing the study questionnaire, participants performed three plank (prone, right and left side) endurance tests in random order, with 1–2 min of recovery between each test. Participants wore their usual athletic clothing and training shoes. Each test was performed on a yoga mat (4 mm thick). For the prone plank (PP) test, participants were instructed to maintain a straight line between shoulders, hip and ankles while supported on their forearms and toes. For right (RSP) and left side (LSP) plank tests, participants were instructed to maintain a straight spine without lateral flexion while on their side supported by that forearm and foot; the top foot placed in front of the bottom one and the top arm was held at their side. For all plank tests, participants were cued to hold their trunk in a neutral position. Consistent with Imai and Kaneoka [22], each test was terminated when the participant was unable to hold any longer or their pelvis moved up or down 5 or more centimeters. One investigator assessed the PP, RSP and LSP for all participants. Hold times for the PP, RSP and LSP tests were recorded in seconds, using a stopwatch up to 120 s, which is the maximum hold time for young healthy adults [23]. Each plank-test time was recorded. The sum of the three plank-test times was also calculated.

Injury Surveillance: During the season, all injuries were recorded and maintained by the school’s athletic training staff by using Athletic Trainer System^®^ software. A lower extremity time-loss injury was defined as a lower-back or lower extremity medical problem resulting from sport participation that required an athlete to be removed from a practice or meet or to miss one or more subsequent practices or meets [24]. Injury date, body location injured, type of injury and days of missed or limited practice and/or competition were recorded. Injuries were classified as acute or overuse. Overuse injuries were those that were sustained with gradual onset and underlying pathogenesis of repetitive microtrauma [25]. Injury data for participants were provided to the primary investigator by the head athletic trainer after the seasons ended.

Statistical Analysis: Independent *t*-tests were used to compare mean baseline characteristics between females and males, as well as to compare the mean PP, RSP and LSP times of athletes who did and did not experience a lower extremity overuse injury during the season. With respect to lateral asymmetry in side plank tests, the absolute difference in times between RSP and LSP was calculated and labeled as RSP/LSP difference. Plank times were also compared between athletes with longer (primary event ≥800 m) and shorter (primary track event ≤400 m and field events) durations. Binomial logistic regression was used to calculate the odds ratios (ORs) and 95% confidence intervals (CIs) to determine the likelihood of lower extremity overuse injury for (1) athletes with plank-test times <60 s (higher likelihood group) compared to ≥60 s (lower likelihood/referent group); (2) athletes with plank-test times of 120 s (lower likelihood/referent group) compared to those <120 s (higher likelihood group); (3) athletes in the lowest (shortest times) quartile for PP, RSP, LSP and summed-plank times (higher likelihood group) compared to only those in the highest (longest times) quartile (lower likelihood group); and (4) athletes in the lowest (shortest times) quartile for summed-plank times (higher likelihood group) compared to those in the 2nd, 3rd and highest (longer times) quartiles (lower likelihood/referent group). Statistical analyses were conducted by using SPSS version 27.0 (IBM; Armonk, NY, USA), with a *p*-value for significance set at <0.05.

## 3. Results

The sample consisted of 29 freshmen, 19 sophomores, nine juniors and 18 seniors, with a similar grade distribution by sex (*p* = 0.52). Of the 75 athletes, 33 were classified as mid-distance or distance runners (primary event ≥800 m), 27 as sprinters (primary event ≤400 m), eight as throwers and seven as jumpers/vaulters. On average, male athletes were significantly taller and heavier (<0.01) (Table 1). Twenty-five athletes (33.3%) experienced a lower extremity time-loss overuse injury during their intercollegiate season. The most common body location injured was the foot/ankle (*n* = 8), followed by the hip/thigh (*n* = 7), shin/calf (*n* = 6) and knee (*n* = 4).

The mean times for the three plank tests, the sum of the plank tests and the difference between RSP and LSP were similar between females and males (*p* > 0.05) (Table 1). The mean plank times for athletes who experienced a lower extremity overuse injury during the season and those who did not incur injury were not significantly different (PP (*p* = 0.83), RSP (*p* = 0.98), LSP (*p* = 0.70) and summed plank time (*p* = 0.83)) (Table 2). No significant differences were found for asymmetry between males and females (*p* = 0.51), or between injured (11.5 ± 14.8 s) and non-injured (9.3 ± 11.7 s) athletes (*p* = 0.48).

When comparing athletes in events of longer and shorter duration, those in longer events were younger, lighter and had lower BMIs (Table 3). On average, athletes in longer duration events had shorter hold times for the prone plank times (<0.001) and summed planks (*p* = 0.02). No significant mean differences were observed between athletes in longer and shorter events for RSP, LSP or RSP/LSP difference (Table 3). Similarly, no significant risk relationships were found between overuse injury and plank times, summed planks or RS/LSP difference for mid-distance/distance runners or sprints/field event athletes. 

Overall, 17.3%, 24.0% and 26.7% of athletes had hold times <60 s for the PP, RSP and LSP, respectively. When comparing athletes with hold times of <60 and ≥60 s by injury status, no significant associations were found for PP (*p* = 0.40), RSP (*p* = 0.16) or LSP (*p* = 0.66) (Table 4.) Among our sample, 44%, 24% and 21.3% held the PP, RSP and LSP for the full 120 s. Risk relationships were not significant between those holding the maximal time on the PP (*p* = 1.0), RSP (*p* = 0.57), LSP (*p* = 0.43) or summed planks (*p* = 0.83) and those with shorter hold times (Table 4). Similarly, no significant risk relationships were found for injury status for athletes in the lowest quartile of hold times (shortest) compared to those in the highest quartile of hold times (longest) for the PP (*p* = 0.62), RSP (*p* = 0.90), LSP (*p* = 0.49) or summed planks (*p* = 0.91), nor for those in the lowest quartile for summed plank times relative to those in the upper three quartiles (*p* = 1.0) (Table 4).

## 4. Discussion

The purpose of this study was to determine whether prone or side plank hold times were associated with lower extremity overuse injury in NCAA cross country and track-and-field athletes. While we hypothesized that shorter plank times would be associated with a higher incidence of lower extremity overuse injury, our results did not support this hypothesis. Our findings are not consistent with those of De Blaiser et al., who observed lower prone and side plank times in students who went on to experience overuse injury [7]. Differences in our findings could be due to different populations studied and differing injury definitions. Their definition included any lower extremity injury that resulted in functional limitation during physical activity or sports [7]. Our findings with respect to side plank times are consistent with those of Leetun et al., who also did not observe a significant relationship between times and lower extremity injury in their sample of 140 collegiate athletes [12].

Side planks require hip abductor and external rotator activity [8]. As increased hip adduction and internal rotator motion have been associated with common running injuries, such as patellofemoral pain [11], it seems plausible that weaker side plank muscles may contribute to overuse-type injuries. However, static holds may not be a valid measure of trunk and hip control during dynamic running impacts, as they do not assess muscle control though a range of motion while in motion [26]. For example, in a study that examined side plank test assessments, hold times for side planks were not correlated to any kinematic changes, including trunk flexion, extension, lateral flexion and rotation, during running that occurred with fatigue [27]. Dynamic isokinetic lower extremity strength values have been linked with running kinematics, while static isometric strength values have not [26].

In the present study, we did not find a significant difference in hold times for any plank type between males and females. This is in contrast to the findings of Leetun et al. [12] and McGill et al. [23] in which females had significantly shorter times for side planks than males. In our sample, the side plank times for females were similar to males. Leetun et al. studied both basketball and cross country athletes, with basketball players making up 75% of their sample, but observed shorter hold times in females regardless of the sport [12]. Our female mean side plank times were longer than their mean of 58.9 s, while our male mean side plank times were shorter than their mean of 84.3 s [12]. The mean plank and side plank times in the present study were about 15 s lower on average than those reported by Imai and Kaneoka in high-school soccer players [22]. The difference in hold times may be because their study did not use a maximum hold time, included only male players and that soccer players may require increased core demands for changes of direction during matches of longer duration than track events. We found that mid-distance/distance runners had shorter prone plank times than sprinters/field event athletes. Although not measured in this study, we speculate that this difference may be because sprint/field event athletes are more likely to participate in weight training or perform movements that involve their core muscles during their in-season and off-season training. As RSP and LSP times were similar between athletes doing longer and shorter events, the difference in summed planks was likely a result of the prone plank. 

Considering that plank exercises require no special equipment, are safe, and programs including them are effective at improving hip- and trunk-strength measures [21], they are often included in rehabilitation programs for injured runners [14,15,16,17,18]. However, whether improved plank times are associated with reduced injury risk in cross country and track-and-field runners is not clear. For example, in a randomized interventional study of over 500 first-time marathon runners, those in the experimental group completed a 10-min workout two or three times per week, which included a plank component [19]. Each workout included about 1 min each of the PP, RSP and LSP, in addition to squats, lunges and toe-touches [19]. They found that runners in the experimental group, which included the plank exercises, did not have a significantly lower incidence of overuse injury during their training or marathon race than those in the observation (control) group [19].

While a major strength of our study was its prospective nature of minimizing measurement bias when assessing the three plank times in uninjured athletes at the start of the season, some limitations of the study are noteworthy. First, our sample size was limited by the number of athletes on the university cross country and track-and-field teams and the lack of support for our hypothesis may have been due to type II error. While most risk relationships were in the expected direction, none reached significance. Second, we limited hold times to 120 s based on normative data and means from prior studies [10,12,23]. However, this may have generated a ceiling affect in the athletes, since, within our sample, 44%, 24% and 21.3% of athletes held the PP, RSP and LSP for the full 120 s potentially lowering the means. Future studies with larger sample sizes may help determine normative values for these sports at the collegiate level and whether there is a longer hold-time cut point that would be meaningful. Third, while hold times for PP have been strongly correlated to EMG activity of abdominal muscles [6], other factors besides the athlete’s trunk-muscle endurance, such as motivation and shoulder discomfort or fatigue, may have influenced hold times. The plank tests are valid measures of endurance, but the endurance necessary for the tests may not reflect that needed for lumbopelvic control during running and field event activities. Lastly, training volume and intensity during the season may have influenced injury risk during the season. However, information for these variables were not available to evaluate their effects on the relationship between plank time and overuse injury.

## 5. Conclusions

Individual and summed plank hold times were not associated with lower extremity overuse injury during the competitive season. Trunk muscle endurance tests in isolation may not be as sensitive as lower extremity injury screening tools in track and cross country athletes, as these tests might not capture the dynamic demands of these sports.

## Figures and Tables

**Table 1:** Baseline characteristics and plank-test times of NCAA cross country and track-and-field athletes.

Variables	Total (*n* = 75)		Females (*n* = 39)		Males (*n* = 36)		*p*-Value *	Effect Size
	Mean	(SD)	Mean	(SD)	Mean	(SD)		Cohen’s d
Age (y)	20.0	(1.3)	20.0	(1.2)	20.1	(1.4)	0.65	0.11
Mass (kg)	66.6	(12.8)	62.2	(12.3)	71.3	(11.8)	<0.01	0.76
Height (m)	1.7	(0.1)	1.7	(0.1)	1.8	(0.1)	<0.01	2.03
BMI (kg/m^2^)	22.1	(3.1)	22.0	(3.2)	22.1	(3.1)	0.96	0.01
Prone plank (s)	90.7	(31.8)	93.4	(28.8)	87.9	(24.9)	0.46	0.17
Right-side plank (s)	75.4	(32.0)	71.5	(29.0)	79.6	(34.9)	0.28	0.25
Left-side plank (s)	74.7	(31.7)	70.9	(31.8)	78.8	(31.5)	0.28	0.25
Summed planks (s)	240.9	(84.8)	235.8	(77.0)	246.3	(93.3)	0.59	0.12
RSP/LSP Difference	10.0	(12.8)	10.9	(13.7)	9.0	(11.8)	0.51	0.15

* Two sample *t*-test of differences of means between females and males.

**Table 2:** Baseline characteristics and plank-test times by lower extremity overuse injury status in NCAA cross country and track-and-field athletes.

Variables	Total (*n* = 75)		Injured (*n* = 25)		Non-Injured (*n* = 48)		*p*-Value *	Effect Size
	Mean	(SD)	Mean	(SD)	Mean	(SD)		Cohen’s d
Age (y)	20.0	(1.3)	19.9	(1.2)	20.1	(1.4)	0.45	0.19
Mass (kg)	66.6	(12.8)	66.6	(15.3)	65.9	(11.3)	0.84	0.05
Height (m)	1.7	(0.1)	1.7	(0.1)	1.7	(0.1)	0.86	0.04
BMI (kg/m^2^)	22.1	(3.1)	21.9	(3.6)	21.9	(2.9)	0.99	<0.01
Prone plank (s)	90.7	(31.8)	90.4	(33.6)	92.1	(31.1)	0.83	0.05
Right-side plank (s)	75.4	(32.0)	76.6	(32.2)	76.4	(31.9)	0.98	<0.01
Left-side plank (s)	74.7	(31.7)	73.2	(31.7)	76.2	(32.4)	0.70	0.09
Summed planks (s)	240.9	(84.8)	240.2	(86.8)	244.7	(84.6)	0.83	0.05
RSP/LSP Difference	10.0	(12.8)	11.5	(14.8)	9.3	(11.7)	0.48	0.17

* Two sample *t*-test of differences of means between injured and non-injured athletes.

**Table 3:** Baseline characteristics and plank-test times by event type in NCAA cross country and track-and-field athletes.

Variables	Total (*n* = 75)		Mid-Distance and Distance Events (*n* = 33)		Sprints and Field Events (*n* = 42)		*p*-Value *	Effect Size
	Mean	(SD)	Mean	(SD)	Mean	(SD)		Cohen’s d
Age (y)	20.0	(1.3)	19.5	(1.2)	20.4	(1.2)	0.002	0.76
Mass (kg)	66.6	(12.8)	61.3	(8.8)	70.8	(14.0)	0.001	0.80
Height (m)	1.7	(0.1)	1.7	(0.1)	1.7	(0.1)	0.80	0.06
BMI (kg/m^2^)	22.1	(3.1)	20.4	(1.8)	23.4	(3.3)	<0.001	1.08
Prone plank (s)	90.7	(31.8)	76.6	(35.4)	101.8	(23.6)	<0.001	0.86
Right-side plank (s)	75.4	(32.0)	71.2	(33.1)	78.8	(31.1)	0.31	0.24
Left-side plank (s)	74.7	(31.7)	67.5	(32.1)	80.4	(30.5)	0.08	0.41
Summed planks (s)	240.9	(84.8)	215.3	(89.8)	260.9	(75.9)	0.02	0.55
RSP/LSP difference	10.0	(12.8)	12.1	(11.5)	8.4	(13.7)	0.21	0.29

* Two sample *t*-test of differences of athletes in longer and shorter duration events.

**Table 4:** Likelihood of lower extremity overuse injury by prone, right- and left-side plank test and summed hold times in NCAA cross country and track-and-field athletes.

	Total (*n* = 75)				Males (*n* = 36)				Females (*n* = 39)			
Classification	*n*	% Inj	OR	95% CI	*n*	% Inj	OR	95% CI	*n*	% Inj	OR	95% CI
Prone plank <60 s	13	46.1	1.7	0.5–5.6	8	37.5	1.5	0.3–7.8	5	60.0	2.4	0.4–16.5
Prone plank ≥60 s	62	33.9	1.0	Ref	28	28.6	1.0	Ref	34	38.2	1.0	Ref
Right-side plank <60 s	18	50.0	2.2	0.7–6.4	7	42.9	2.0	0.4–10.8	11	54.5	2.2	0.5–8.9
Right-side plank ≥60 s	57	31.6	1.0	Ref	29	27.6	1.0	Ref	28	35.7	1.0	Ref
Left-side plank <60 s	20	40.0	1.3	0.4–3.6	8	37.5	1.5	0.3–7.8	12	41.7	1.0	0.3–4.1
Left-side plank ≥60 s	55	34.5	1.0	Ref	28	28.6	1.0	Ref	27	40.7	1.0	Ref
Prone plank <120 s	42	33.3	1.0	0.4–2.6	20	30.0	0.9	0.2–3.9	22	36.4	1.0	0.3–3.9
Prone plank 120 s	33	33.3	1.0	Ref	16	31.3	1.0	Ref	17	35.3	1.0	Ref
Right-side plank <120 s	57	31.6	0.7	0.2–2.2	24	25.0	0.5	0.1–2.0	33	36.4	1.1	0.2–7.2
Right-side plank 120 s	18	38.9	1.0	Ref	12	45.5	1.0	Ref	6	33.3	1.0	Ref
Left-side plank <120 s	59	35.6	1.7	0.5–5.8	27	29.6	0.8	0.2–4.2	32	40.6	4.1	0.4–38.2
Left-side plank 120 s	16	25.0	1.0	Ref	9	33.3	1.0	Ref	7	14.3	1.0	Ref
Summed plank <360 s	62	33.9	1.7	0.5–6.3	28	28.6	0.7	0.1–3.5	34	38.2	2.5	0.2–24.6
Summed plank 360 s	13	30.8	1.0	Ref	8	37.5	1.0	Ref	5	20.0	1.0	Ref
Prone plank in lowest quartile (≤69 s)	20	40.0	1.3	0.4–4.2	11	36.4	1.3	0.2–6.4	9	44.4	1.5	0.3–7.6
Prone plank in highest quartile (120 s)	33	33.3	1.0	Ref	16	31.3	1.0	Ref	17	35.3	1.0	Ref
Right-side plank in lowest quartile (<60 s)	18	38.9	1.1	0.3–4.1	7	42.9	1.1	0.2–6.9	11	36.4	1.4	0.2–11.1
Right-side plank in highest quartile (≥115 s)	19	36.8	1.0	Ref	12	41.7	1.0	Ref	7	28.6	1.0	Ref
Left-side plank in lowest quartile (≤54 s)	19	36.8	1.6	0.4–6.5	7	42.9	2.0	0.3–14.8	12	33.3	1.5	0.2–11.1
Left-side plank in highest quartile (≥102 s)	19	26.3	1.0	Ref	11	27.3	1.0	Ref	8	25.0	1.0	Ref
Summed plank time in lowest quartile (≤189 s)	18	33.3	1.1	0.3–4.3	7	16.7	0.9	0.1–6.1	11	27.3	2.6	0.2–31.4
Summed plank time in highest quartile (≥311 s)	19	31.6	1.0	Ref	11	27.8	1.0	Ref	8	12.5	1.0	Ref
Summed plank time in lowest quartile (≤189 s)	18	33.3	1.0	0.3–3.1	7	42.9	2.0	0.4–10.8	11	27.3	0.6	0.1–2.7
Summed plank time in 2nd, 3rd and 4th quartiles (>190 s)	57	33.3	1.0	Ref	29	27.6	1.0	Ref	28	39.3	1.0	Ref

*n*, number at risk; % inj, percentage injured within group; OR, odds ratio; CI, confidence interval, Ref, reference group.

## Data Availability

The data presented in this study are available upon request from the corresponding author.
